# Phenylketonuria in Saudi Arabia: An Overview of Diagnosis, Genetics, and Therapeutic Strategies

**DOI:** 10.3390/biology15141122

**Published:** 2026-07-10

**Authors:** Faris J. Tayeb, Rashid Mir, Sael Alatawi

**Affiliations:** 1 Department of Medical Laboratory Technology, Faculty of Applied Medical Sciences, University of Tabuk, Tabuk 71491, Saudi Arabia; s.alatawi@ut.edu.sa; 2 Prince Fahd Bin Sultan Research Chair for Biomedical Research, University of Tabuk, Tabuk 71491, Saudi Arabia

**Keywords:** phenylketonuria, PKU, phenylalanine hydroxylase, PAH gene, Saudi Arabia, newborn screening, sapropterin, BH4, gene therapy, consanguinity, pediatric metabolic disease, genome-wide association studies, GWAS

## Abstract

Phenylketonuria (PKU) is an autosomal recessive inborn error of phenylalanine (Phe) metabolism caused by pathogenic variants in the phenylalanine hydroxylase (PAH) gene, resulting in toxic phenylalanine accumulation that, if untreated, causes profound intellectual disability and neurodevelopmental impairment. PKU is especially significant in the Kingdom of Saudi Arabia (KSA), where high consanguinity rates substantially elevate disease prevalence relative to Western populations, and the country’s expanding newborn screening programs have highlighted PKU as a persistent public health concern. This review provides a translational synthesis of the Saudi PKU literature, covering epidemiology, molecular pathophysiology, genetics, clinical presentation, diagnosis, treatment, prognosis, and future directions. We summarize global and regional incidence data and show that Saudi Arabia, driven by consanguinity, is among the countries with the highest reported PKU burden worldwide. We review the metabolic basis of phenylalanine neurotoxicity and the allelic heterogeneity of the PAH gene, with attention to variants enriched in Saudi and Arab cohorts, including the founder allele p.R252W. Diagnostic pathways anchored in newborn screening and tandem mass spectrometry are discussed alongside neurodevelopmental outcomes and gaps in Saudi PKU surveillance. We outline established and emerging therapies, including dietary management, sapropterin (BH4), pegvaliase, large neutral amino acids, and investigational gene and mRNA therapies. Throughout, we identify where genuine Saudi-specific evidence exists and where general PKU knowledge is extrapolated to the Saudi context because of limited local data, most notably the absence of a national PKU registry. This review is intended to serve as a translational reference for clinicians, metabolic dietitians, geneticists, and policymakers engaged in PKU care in Saudi Arabia and the wider Arab region.

## Simple Summary

Phenylketonuria (PKU) is an autosomal recessive inborn error of phenylalanine (Phe) metabolism caused by pathogenic variants in the phenylalanine hydroxylase (PAH) gene. In the Kingdom of Saudi Arabia, the management of PKU has advanced dramatically with the adoption of national screening programs. The Saudi government began a premarital screening program in 2003 and a comprehensive national newborn screening (NBS) program in 2005. Although the frequency of PKU in Saudi Arabia is thought to be around 1 in 14,623 births, it is still a major concern for the King Faisal Specialist Hospital and Research Center and the Saudi Society for Medical Genetics. Because the ailment is autosomal recessive, the high prevalence of parental consanguinity in the region is the key risk factor for the transmission of the condition. This review summarizes the Saudi PKU literature with a translational focus, covering epidemiology, molecular pathophysiology, genetics, diagnosis, treatment, prognosis, and future directions.

## 1. Introduction

Phenylketonuria (PKU; OMIM #261600) is one of the most well-characterized inborn errors of amino acid metabolism. It results from a deficiency of the hepatic enzyme phenylalanine hydroxylase (PAH), which normally converts phenylalanine to tyrosine [1,2]. Loss of PAH activity leads to phenylalanine accumulation in blood and tissues, which has particularly severe consequences for a developing brain. If left untreated, classic PKU causes profound intellectual disability, behavioral disturbance, epilepsy, and movement disorder. The condition is inherited in an autosomal recessive pattern, with pathogenic variants distributed across the PAH gene on chromosome 12q23.2.

PKU occupies an important place in the history of medicine as one of the first genetic conditions shown to be preventable through early diagnosis and dietary intervention. Asbjørn Fölling’s discovery in 1934, followed in the 1960s by the development of a low-phenylalanine diet and Robert Guthrie’s bacterial inhibition assay for newborn screening [3], established a model for translational metabolic medicine that still shapes the modern development of precision therapies. Contemporary PKU management now extends well beyond dietary restriction to include enzyme cofactor therapy with sapropterin dihydrochloride (Kuvan^®^), enzyme substitution with pegvaliase (Palynziq^®^; BioMarin Pharmaceutical, San Rafael, CA, USA), and a pipeline of gene- and mRNA-based therapies currently under clinical investigation [4].

In Saudi Arabia, awareness of PKU as a public health problem grew in the 1980s, alongside the broader regional expansion of newborn screening programs. This growth was driven by the recognition that high consanguinity rates substantially increase the burden of autosomal recessive disorders in the Saudi population [5,6]. Early molecular studies identified PAH variants in Saudi and Arab cohorts that differed from those predominant in European populations, highlighting that the Saudi mutation spectrum has distinct regional features [1,2]. The Saudi National Newborn Screening Program, administered through the Ministry of Health, has expanded progressively since the 1980s and now provides nationwide PKU coverage; however, a nationally representative registry linking genotype, phenotype, BH4 responsiveness, and long-term neurodevelopmental outcomes does not yet exist. This gap is the main motivator of this review [6,7].

In Saudi Arabia, PKU is a disease of clear epidemiological and clinical importance. The country’s high consanguinity rate, estimated at 50–60% of marriages, substantially increases the prevalence of autosomal recessive disorders, including PKU [5]. Although national newborn screening programs have been in operation since the 1980s [6], published data on incidence, the PAH mutation spectrum, BH4 responsiveness, long-term neurodevelopmental outcomes, and real-world treatment patterns remain limited [7]. This gap matters clinically because the genetic architecture of PKU in the Saudi population is likely to differ from that seen in European or East Asian cohorts, with implications for genotype-specific therapy selection, dietary management, and precision counseling. This review addresses that gap by providing a translational synthesis of PKU epidemiology, metabolic mechanisms, PAH genetics, clinical presentation, diagnostic approaches, neurodevelopmental outcomes, and therapeutic strategies in the Saudi healthcare context. It also identifies key research priorities and policy goals for improving outcomes in Saudi children and adults living with PKU.

This review is distinct from and complementary to two existing publications that might otherwise appear to cover a similar topic. El-Metwally et al. [8] conducted a systematic review focused on PKU prevalence and incidence across Arab countries, Turkey, and Iran using screening-program data; however, the scope of this study is epidemiological and does not extend to the PAH mutation spectrum, clinical presentation, diagnostic pathways, or therapeutic strategies. Elhawary et al. [9] provide a broad narrative review of PKU genetics, pathophysiology, and management with a global scope; their synthesis is not regionally focused and does not examine the Saudi-specific genetic architecture, local newborn-screening landscape, or Saudi treatment-access barriers in depth. The present review differs from both by anchoring the discussion of epidemiology, genetics, and health systems in the Saudi context while also providing a full overview of PKU’s pathophysiology, clinical course, diagnosis, and current and emerging therapies. It also clarifies where Saudi-specific evidence exists and where general PKU knowledge is being applied to the Saudi setting because of limited local data.

## 2. Epidemiology of PKU in Saudi Arabia

Globally, the PKU incidence varies widely by ethnicity and geographic region, ranging from about 1 in 2600 to 1 in 333,000 live births. A large meta-analysis of newborn-screening data pooled this country-level variation into a global estimate of approximately 6.0 per 100,000 live births (95% CI: 5.07–6.93), with rates ranging from 0.3 per 100,000 in Thailand to 38.1 per 100,000 in Turkey [10]. In Europe, the estimated incidence is about 1 in 10,000 to 1 in 15,000, with the highest rates reported in Turkey, Ireland, and Eastern Europe. In East Asian populations, the incidence is generally lower. In the Middle East and the Arabian Peninsula, the PKU incidence is substantially higher, largely because consanguineous marriage increases the chance that both parents carry the same recessive PAH variant [8]. In Saudi Arabia, published estimates of PKU incidence range from 1 in 4000 to 1 in 6000 live births, or roughly 18–24 per 100,000, placing the Kingdom among the countries with the highest PKU burden worldwide [11] [Table 1]. Data from the Saudi National Newborn Screening Program have confirmed consistently elevated detection rates since the program expanded in the 1990s, with tandem mass spectrometry-based expanded screening playing a major role in this improved detection performance [11,12]. However, a nationally representative registry with systematic genotype data, regional breakdowns, and longitudinal outcome tracking does not yet exist, leaving a major gap in the epidemiological evidence base.

Consanguinity is the main demographic driver of the elevated PKU prevalence in Saudi Arabia. When two carriers of a pathogenic PAH variant marry, each pregnancy carries a 25% chance of producing an affected child, a risk that rises sharply in populations where first- and second-cousin marriages are common. The frequency of PAH variant carriers in Saudi Arabia has not been accurately established, but is thought to be considerably higher than the 1 in 50 figure reported in European populations. Certain PAH variants may also be regionally enriched through founder effects, consistent with patterns described in other consanguineous populations such as in Turkey, Iran, and Pakistan. Maternal PKU is an under-recognized epidemiological concern in Saudi Arabia. Women with PKU with poorly controlled phenylalanine levels who become pregnant are at high risk of delivering children with microcephaly, congenital heart defects, intellectual disability, and facial dysmorphism, collectively termed maternal PKU syndrome [13]. Given the elevated PKU prevalence in Saudi Arabia and the possibility that diagnosis may be lost to follow-up after childhood, systematic pre-conception metabolic screening and counseling for women of reproductive age with PKU should be treated as an important and under addressed public health priority [14].

## 3. Pathophysiology and Metabolic Mechanisms

**Figure 1:**
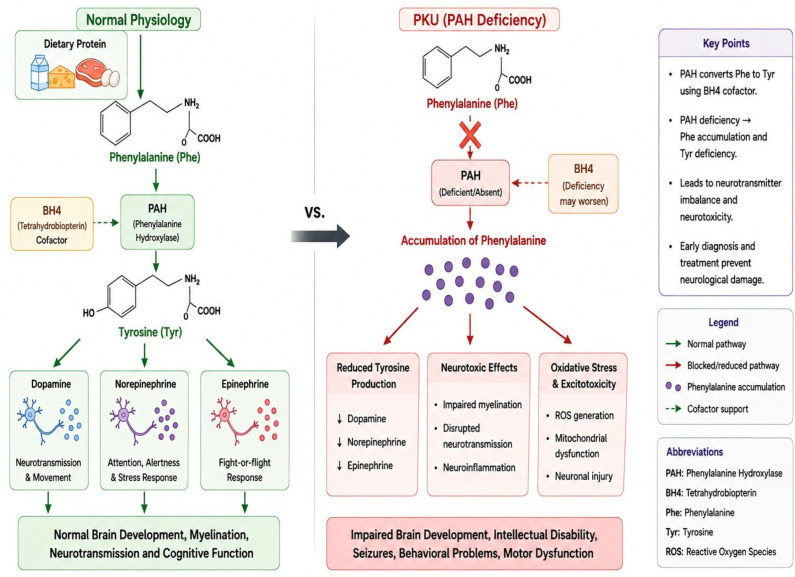
Phenylalanine metabolism pathway and consequences of PAH deficiency in PKU. Original schematic conceptualized by the authors and rendered with the assistance of ChatGPT-4o (OpenAI, San Francisco, CA, USA); no third-party stock or licensed image assets were used. The authors reviewed and verified the figure for scientific accuracy.

Phenylalanine hydroxylase (PAH) is a homotetrameric enzyme expressed almost exclusively in hepatocytes, but a small amount is expressed in the kidneys and small intestine. Its principal function is the hydroxylation of L-phenylalanine to L-tyrosine—the rate-limiting step in phenylalanine catabolism—using tetrahydrobiopterin (BH4) and molecular oxygen as cofactors [15]. Each catalytic cycle oxidizes BH4 to dihydrobiopterin (BH2), which is then reduced back to BH4 by dihydropteridine reductase (DHPR). This regeneration cycle is essential for sustained PAH activity, and impairment of DHPR or BH4 biosynthesis produces a phenocopy of PAH-deficient PKU (BH4 deficiency syndromes) that requires distinct management [16] as depicted in [Figure 1].

When PAH activity is severely reduced or absent, dietary phenylalanine is not converted to tyrosine and instead accumulates in blood. Some of it is shifted into minor alternative pathways, producing phenylpyruvate, phenylacetate, and phenyllactate, the ‘phenylketones’ that give the condition its name, which are excreted in urine. The biochemical hallmark of untreated classic PKU is a plasma phenylalanine concentration above 1200 µmol/L (normal < 120 µmol/L). Simultaneously, tyrosine becomes depleted, reducing the biosynthesis of dopamine, adrenaline, noradrenaline, and melanin. The neurotoxicity of excess phenylalanine is multifactorial and still not completely understood. The most prominent mechanism is competitive inhibition of the large neutral amino acid transporter (LAT1) at the blood–brain barrier, where Phe competes with other large neutral amino acids (LNAAs) including tyrosine, tryptophan, leucine, isoleucine, valine, and methionine. Because LAT1 operates under competitive substrate saturation, elevated plasma Phe suppresses brain uptake of these amino acids and leads to a global deficiency of neurotransmitter precursors in the central nervous system, most critically serotonin (from tryptophan) and dopamine (from tyrosine) [17].

Additional mechanisms of neurotoxicity include impaired myelination through disruption of oligodendrocyte function; abnormal dendritic arborization and synaptogenesis due to disturbed neurotrophic signaling; oxidative stress from imbalanced redox metabolism; and inhibition of key cerebral enzymes including pyruvate kinase and glutamate decarboxylase [18]. The net effect is a diffuse encephalopathy that is most damaging during periods of rapid brain development, including fetal development, infancy, and early childhood, but can continue to affect cognitive and neuropsychiatric function throughout adulthood if phenylalanine control is inadequate.

In BH4 deficiency disorders, the metabolic consequences extend beyond PAH insufficiency. Reduced BH4 also decreases the activity of tyrosine hydroxylase and tryptophan hydroxylase, enzymes critical for dopamine and serotonin synthesis, as well as nitric oxide synthase. This broader metabolic disruption explains why BH4 deficiency syndromes present with more severe and progressive neurological features, including movement disorders, hypotonia, seizures, and autonomic dysfunction [19], and why dietary Phe restriction alone is not sufficient therapy in these conditions.

## 4. Genetics of PKU

**Figure 2:**
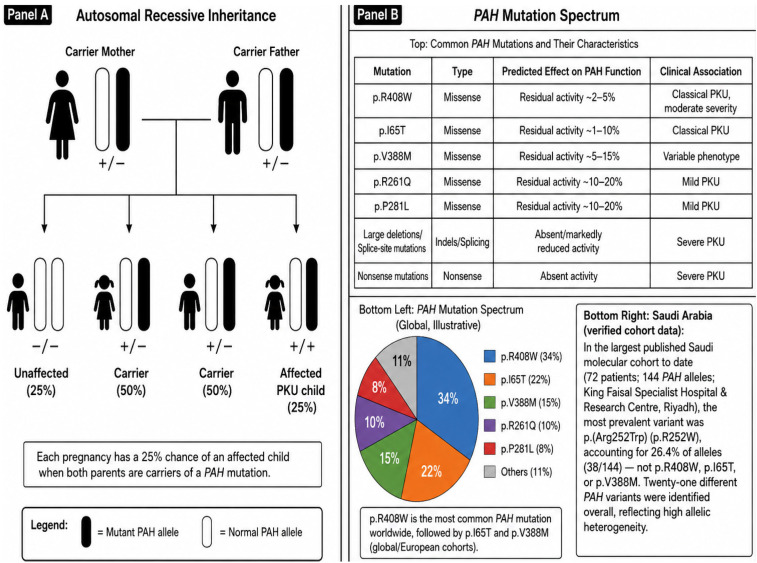
Inheritance pattern and PAH mutation spectrum in PKU. Original schematic created by the authors for this review; mutation-spectrum percentages are illustrative of patterns reported predominantly in European cohorts (where p.R408W is most prevalent) and do not represent a globally uniform distribution, as allele frequencies differ substantially in East Asian, Middle Eastern, and other non-European populations.

The PAH gene, located on chromosome 12q23.2, spans about 90 kb of genomic DNA and encodes a protein of 452 amino acids. More than 1000 pathogenic and likely pathogenic variants have been cataloged in the PAHvdb database (www.biopku.org), encompassing missense mutations (the majority), nonsense mutations, small insertions and deletions, splice-site variants, and large structural rearrangements [20] [Figure 2]. The remarkable allelic heterogeneity of the PAH gene is a defining feature of PKU genetics and poses a major challenge for genotype–phenotype correlation [21], because even functionally similar variants can produce different clinical phenotypes depending on their specific effects on enzyme folding, stability, and cofactor binding.

PKU follows strict autosomal recessive inheritance. Heterozygous carriers are phenotypically unaffected and usually have normal or near-normal phenylalanine levels. Phenotype severity is mainly determined by residual PAH enzyme activity: classic PKU (residual activity < 1%) requires lifelong dietary restriction; moderate PKU (1–5% activity) has intermediate dietary requirements; and mild hyperphenylalaninemia (MHP; >5% activity) may require limited or no dietary intervention [22]. In practice, the plasma phenylalanine concentration at diagnosis and response to BH4 loading are used as functional surrogates for genotype-based prediction.

In Saudi Arabian and broader Arab populations, several PAH variants are present at higher frequencies than in European cohorts. Among these, p.R252W is the most prevalent variant reported to date, accounting for 26.4% of alleles (38/144) in the largest published Saudi cohort [23,24]. The p.R261Q missense variant, associated with moderate PKU and BH4 responsiveness, has been reported in multiple Saudi and Arab PKU cohorts and may represent a founder allele [23,24] [Figure 2]. The splice-site variant IVS10-11G>A, which causes exon skipping and loss of function, has also been identified in Middle Eastern patients. Novel private variants, i.e., pathogenic mutations not previously described in international databases, are disproportionately common in consanguineous Saudi families [24,25], where affected children are often homozygous for rare variants that may be geographically restricted. Whole-exome sequencing has therefore become an important adjunct to PAH gene panel testing in families in which standard sequencing has only identified one pathogenic variant or none at all [9].

Genotype–phenotype correlation analysis in PKU is imperfect but clinically useful. Variants that abolish PAH activity, such as nonsense mutations, frameshift variants, and structural deletions, reliably produce classic PKU, while missense variants that reduce but do not eliminate PAH activity produce a spectrum of phenotypes [Table 2]. BH4 responsiveness, defined as a reduction in plasma phenylalanine of at least 30% after a standardized sapropterin loading test, is strongly associated with specific missense variants, particularly those in the regulatory or cofactor-binding domains of PAH where BH4 can stabilize the misfolded protein [26]. In Saudi patients, the frequency of BH4-responsive variants relative to classic-severity variants has not been systematically studied, leaving an critical knowledge gap with direct therapeutic implications.

From a population genetics perspective, the high consanguinity rate in Saudi Arabia increases the frequency of homozygous genotypes. This makes genotype–phenotype prediction easier in affected individuals because homozygotes carry two copies of the same allele, and it also means that rare recessive variants can reach detectable frequencies within geographically or tribally isolated communities. Tribal endogamy, which is common in parts of the Kingdom, may generate regional founder effects that differ from the national allele distribution. A systematic national PAH variant database, similar to those established in Turkey (BioTurkPKU) and Iran, would be of substantial value for counseling, screening strategy, and precision medicine in Saudi Arabia.

## 5. Clinical Presentation and Diagnosis

### 5.1. Clinical Presentation

In countries with functioning newborn screening programs, most PKU patients are identified before symptoms appear, and thus the classic phenotype of untreated PKU is now rarely seen in practice. Even so, understanding the natural history of untreated PKU remains essential for late-presenting or under-screened patients, for interpreting the consequences of metabolic decompensation in treated patients, and for understanding the biological mechanisms of Phe neurotoxicity.

**Figure 3:**
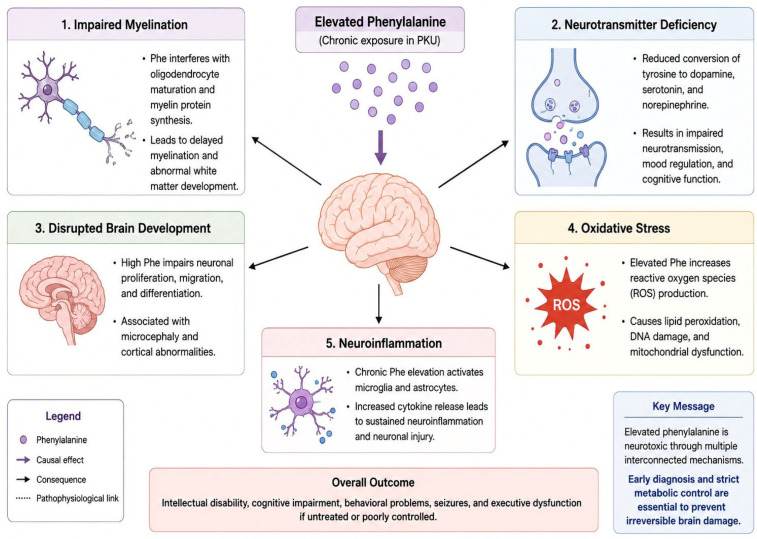
Neurological consequences of elevated phenylalanine in PKU. Original schematic conceptualized by the authors and rendered with the assistance of ChatGPT-4o (OpenAI, San Francisco, CA, USA); no third-party stock or licensed image assets were used. The authors reviewed and verified the figure for scientific accuracy.

Infants with undiagnosed PKU appear clinically normal at birth because maternal PAH activity clears excess phenylalanine during fetal life. In the first weeks after birth, as dietary protein intake increases, plasma phenylalanine rises [Figure 3]. Without treatment, affected infants develop progressive intellectual disability, with IQ scores often below 50; behavioral abnormalities including hyperactivity, aggression, and stereotypies; epilepsy (reported in approximately 25–50% of untreated cases) [27]; eczematous skin rashes related to elevated phenylpyruvate; a characteristic musty or mousy body odor from phenylacetate excretion; and fairer pigmentation relative to unaffected family members due to reduced melanin production caused by tyrosine deficiency [1].

In treated patients, the clinical picture is substantially improved but not completely normalized. Children with early-diagnosed and diet-treated PKU generally achieve IQ scores within the normal range, but they often score somewhat below population means, with particular weaknesses in executive function, attention, working memory, processing speed, and visuospatial ability. Behavioral problems, anxiety, depression, and social difficulties are also common, even in patients with good metabolic control [28]. Neurological complications, including white matter changes on MRI, seen as T2/FLAIR hyperintensities predominantly in the periventricular and posterior white matter, occur in a certain proportion of treated patients and are correlated with phenylalanine excursions above the therapeutic target range.

In adults with PKU, the clinical focus shifts toward chronic neuropsychiatric morbidity, bone health, maternal PKU management, and the challenge of sustaining dietary adherence, which typically declines over adolescence and young adulthood. Poorly controlled adult PKU is associated with progressive cognitive decline, especially in executive function and processing speed; chronic anxiety and depression; reduced bone mineral density related to restricted protein and micronutrient intake [29]; and, in pregnant women, the serious teratogenic consequences of maternal hyperphenylalaninemia discussed above.

### 5.2. Diagnosis

The diagnosis pipeline for PKU begins with newborn blood spot screening using tandem mass spectrometry (MS/MS), which measures plasma phenylalanine and the Phe:Tyr ratio from a dried blood spot collected at 48–72 h of life [Table 3]. A positive screen requires confirmatory plasma amino acid quantification by high-performance liquid chromatography or MS/MS on a venous sample. The diagnosis is confirmed if plasma phenylalanine exceeds 120 µmol/L in the absence of BH4 deficiency. Phenotype classification—classic PKU (Phe > 1200 µmol/L), moderate PKU (Phe 600–1200 µmol/L), mild PKU (Phe 360–600 µmol/L), or mild hyperphenylalaninemia (Phe 120–360 µmol/L)—is based on the untreated phenylalanine concentration and is broadly correlated with genotype and dietary requirements.

BH4 deficiency must be excluded in every newly diagnosed patient, because failure to do so can lead to treatment with dietary restriction alone [30], which is inadequate for BH4-deficient disorders that require neurotransmitter precursor supplementation and specific cofactor therapy [31]. BH4 deficiency is excluded by the urinary pterin profile (elevated neopterin, low biopterin in PTPS deficiency, and elevated biopterin in DHPR deficiency) and, when indicated, measurement of dihydropteridine reductase (DHPR) enzyme activity in erythrocytes.

For patients with confirmed PAH-deficient PKU, BH4 responsiveness testing is recommended to identify candidates for sapropterin therapy. In the standard BH4 loading test, sapropterin is administered at 20 mg/kg/day for 24 h in neonates or for 48 h to 4 weeks in older infants, children, adolescents, and adults [32]; a reduction in plasma phenylalanine of at least 30% from baseline is generally accepted as the threshold for a positive response [33]. Responsiveness strongly predicts benefit from long-term sapropterin therapy and is associated with specific PAH genotypes [34]. In Saudi Arabia, systematic BH4 loading test data across the PKU population have not been published, and the proportion of BH4-responsive patients in this genetically distinct cohort remains unknown.

**Table 3:** Clinical presentation and diagnostic tests. Compiled from published guidelines and the primary literature [31,35].

Significance	Threshold/Finding	Clinical Features/Test	Domain
First-line; enables pre-symptomatic diagnosis	Phe > 120 µmol/L (screen positive)	Tandem mass spectrometry (MS/MS)—Phe spot	Newborn Screening
Defines PKU subtype and dietary urgency	Classic: Phe > 1200 μmol/L; Moderate: 600–1200; Mild: 360–600; MHP: 120–360	Plasma amino acid quantification	Confirmatory Biochemistry
Essential; BH4 deficiency requires different Tx	Biopterin/neopterin ratio; DHPR < normal	Urine pterin profile; DHPR enzyme activity	BH4 Deficiency Exclusion
Identifies candidates for sapropterin therapy	>30% Phe reduction = responsive	Sapropterin 20 mg/kg; Phe measured at 24 h (neonates) to 4 weeks (older patients) [32]	BH4 Loading Test
Late-diagnosed or uncontrolled PKU	White matter hyperintensities; epileptiform activity	EEG, brain MRI (T2/FLAIR)	Neurological Assessment
Monitor treatment adequacy	IQ < 85; executive dysfunction in untreated	IQ testing, Vineland, BRIEF, developmental scales	Neurodevelopment
Restricted diet may compromise bone mineral density	Low BMD; nutritional deficiency	DEXA scan; Vitamin D, calcium, phosphate	Bone Health
Common even in treated patients	Elevated ADHD, anxiety symptoms	ADHD rating scales; anxiety/depression tools	Psychiatric Screening
Guides prognosis, BH4 response, family counseling	Pathogenic variants; genotype classification	PAH gene sequencing (Sanger or NGS/WES)	Genetic Testing

PAH gene sequencing—via Sanger sequencing of the 13 exons and flanking intronic regions, or increasingly by a next-generation sequencing (NGS) panel or whole-exome sequencing (WES)—provides genotype information that refines phenotype prediction, predicts the BH4 response likelihood, and enables accurate recurrence-risk counseling and cascade testing of at-risk relatives [35]. WES is particularly valuable in Saudi families in which standard PAH sequencing yields an incomplete molecular diagnosis, which occurs more often when novel or private variants are present.

### 5.3. Saudi Case-Level Evidence

**Figure 4:**
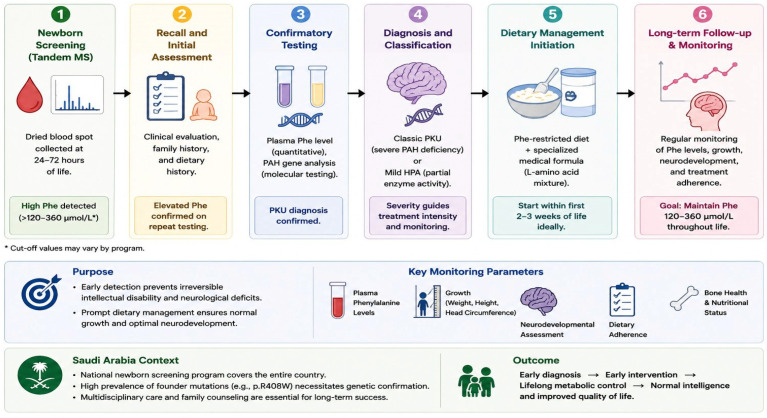
Diagnostic workflow for pediatric PKU in Saudi Arabia. Original schematic created by the authors for this review.

Published Saudi clinical case-level data remain limited to isolated reports rather than systematic case series, but the available accounts illustrate how PKU can present in this population outside routine newborn screening [Figure 4]. Alghamdi et al. described an intellectually normal 32-year-old Saudi woman who presented with recurrent pregnancy loss and two neonatal deaths with congenital heart disease, microcephaly, and intrauterine growth restriction; she was later found, through exome sequencing, to have unsuspected classical PKU (plasma phenylalanine 1642 µmol/L). This case illustrates how maternal PKU syndrome can be the first clinical clue in undiagnosed adult cases [36]. It also highlights the diagnostic vigilance needed for women presenting with unexplained recurrent pregnancy loss in high-consanguinity settings and reinforces the maternal PKU concern raised in Section 2. Such reports are still too few to systematically characterize the Saudi clinical spectrum, and this remains a notable evidence gap.

## 6. Prognosis and Neurodevelopmental Outcomes

The prognosis of PKU is determined mainly by age at diagnosis, the stringency and duration of metabolic control, and the genotype-driven biological severity of the condition. In patients diagnosed through newborn screening and treated from the first weeks of life, intellectual outcomes are overwhelmingly favorable, and most achieve IQ scores within the normal range. However, subtle cognitive deficits still persist in a considerable proportion, particularly deficits in executive functions mediated by the prefrontal cortex, including working memory, cognitive flexibility, sustained attention, inhibitory control, and planning [37].

Neuroimaging abnormalities are detected in a substantial proportion of treated PKU patients, most commonly as symmetric white matter hyperintensities on T2-weighted or FLAIR MRI sequences in the posterior periventricular regions [38]. The clinical significance of these findings is debated: they are not always correlated with cognitive impairment, but their presence is associated with a higher lifetime phenylalanine burden and may indicate subclinical myelin instability. Longitudinal data suggest that white matter changes can be at least partly reversible with improved metabolic control [39], providing a neuroradiological biomarker of treatment adequacy [40].

Psychiatric comorbidity is an increasing area of concern in PKU research. Studies consistently report elevated rates of anxiety disorders, depression, ADHD, and autism spectrum features in PKU patients relative to population norms [41], findings that persist even in patients with lifelong dietary treatment and good metabolic control. This suggests that neuropsychiatric risk in PKU is not explained solely by phenylalanine excess but may also reflect tyrosine depletion, with downstream effects on dopaminergic and noradrenergic signaling; the burden of chronic dietary management; psychosocial stress; and, potentially, genetic modifiers [42].

In Saudi Arabia, long-term neurodevelopmental outcome data for PKU patients are scarce. Published studies from the Kingdom largely focus on screening program performance and biochemical phenotyping, rather than cognitive or psychiatric outcomes. Establishing a longitudinal national PKU cohort with standardized neurodevelopmental assessments—including IQ testing, executive function batteries, psychiatric screening, and neuroimaging—would be a major advance in understanding the real-world burden of PKU in Saudi Arabia and in evaluating the effectiveness of current therapeutic strategies.

The prognosis of untreated or late-diagnosed PKU in Saudi Arabia is more negative and is particularly relevant in communities with limited healthcare access or where newborn screening follow-up fails. Late-presenting classic PKU with established intellectual disability remains a clinical and ethical challenge: dietary restriction and pharmacological therapy can prevent further phenylalanine-mediated damage, but they cannot reverse established neurological injury. Early and sustained treatment is therefore the essential foundation of a favorable prognosis.

## 7. Therapeutic Strategies

**Figure 5:**
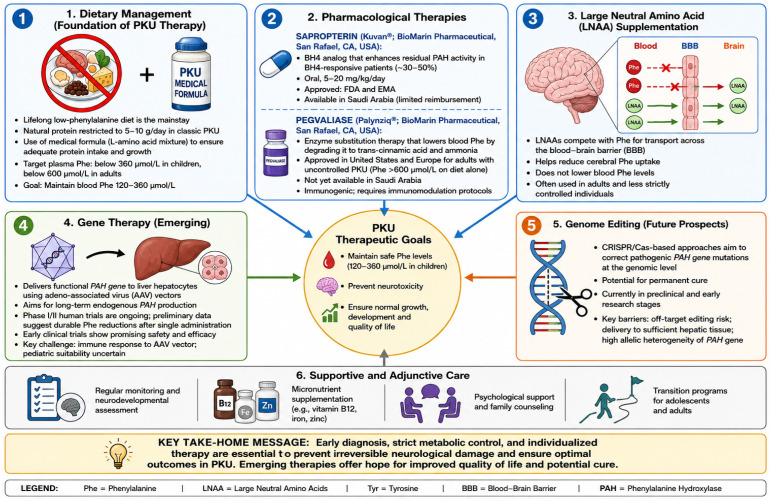
Current and emerging therapeutic strategies for PKU. Original schematic conceptualized by the authors and rendered with the assistance of ChatGPT-4o (OpenAI, San Francisco, CA, USA); no third-party stock or licensed image assets were used. The authors reviewed and verified the figure for scientific accuracy.

The cornerstone of therapeutic management for PKU remains a strict, lifelong low-phenylalanine diet, which restricts natural protein intake and utilizes specialized medical formulas to provide essential amino acids, vitamins, and minerals without the toxic buildup of Phe. Beyond dietary restriction, pharmacological strategies have emerged to enhance metabolic control as depicted in Table 3 and Figure 5.

### 7.1. Dietary Management

The cornerstone of PKU management is a phenylalanine-restricted diet supplemented with a phenylalanine-free amino acid formula to prevent protein deficiency and ensure adequate intake of tyrosine, essential amino acids, vitamins, and minerals. Dietary management must begin within the first weeks of life to prevent intellectual disability; current international guidelines recommend maintaining plasma phenylalanine below 360 µmol/L in children and below 600 µmol/L in adults, although targets are individualized according to age, severity, tolerance, and clinical response [43].

The practical burden of dietary management in PKU is substantial. Natural protein intake must be restricted to about 5–10 g/day in classic PKU, requiring the elimination of high-protein foods including meat, fish, dairy, eggs, legumes, and most grains [44]. Natural phenylalanine tolerance—the maximum daily Phe intake compatible with target blood levels—is determined empirically for each patient and increases progressively with age and body weight [45]. The amino acid formula, which provides most of the daily protein, is often unpalatable, and adherence declines markedly through adolescence and adulthood [46]. In Saudi Arabia, medical formulas are predominantly imported, with limited availability of local alternatives and variable reimbursement by region [47], creating an access gap that directly affects adherence and outcomes.

Glycomacropeptide (GMP)-based formulas are an alternative protein source derived from cheese whey that are naturally low in phenylalanine and generally more palatable than amino acid formula [48]. GMP-based medical foods are increasingly used in high-income countries but remain largely unavailable in Saudi Arabia [49]. Large neutral amino acid (LNAA) supplementation, which works by competitively blocking Phe transport across the blood–brain barrier rather than lowering blood Phe levels, is sometimes used as a dietary adjunct in older patients with poor adherence, although evidence of cognitive benefit remains limited [50].

### 7.2. Pharmacological Therapies

The available therapeutic options and their indications are summarised in Table 4. Sapropterin dihydrochloride (Kuvan^®^; BioMarin Pharmaceutical, San Rafael, CA, USA), the synthetic form of the natural PAH cofactor tetrahydrobiopterin (BH4), is approved by the FDA and EMA for PKU patients with demonstrated BH4 responsiveness. Administered orally at 5–20 mg/kg/day, sapropterin stabilizes the misfolded PAH enzyme [51], restores partial catalytic activity, and allows for dietary phenylalanine liberalization in responsive patients [52]. Responsiveness is mutation-dependent: patients with missense variants that retain partial PAH protein are more likely to respond than those with null alleles. In Saudi Arabia, sapropterin has received regulatory approval, but reimbursement and access remain inconsistent, and systematic BH4 loading test data for Saudi patients are lacking, which limits precision prescription [53].

Pegvaliase (Palynziq^®^; BioMarin Pharmaceutical) is a PEGylated recombinant phenylalanine ammonia lyase (PAL) enzyme derived from the cyanobacterial species Anabaena variabilis. Administered by subcutaneous injection, pegvaliase converts phenylalanine to ammonia and trans-cinnamic acid, thereby bypassing the deficient PAH enzyme entirely [54]. Mechanistically, this differs from classic enzyme replacement therapy: instead of supplying a functional copy of human PAH, pegvaliase uses a non-human enzyme that clears phenylalanine through a separate, BH4-independent pathway, which is why it is more accurately described as enzyme substitution therapy. It can produce dramatic reductions in phenylalanine, often to below-normal levels, and can allow for substantial dietary liberalization in patients who reach maintenance dosing [55]. Pegvaliase is approved in the United States and Europe for adults with uncontrolled PKU (Phe > 600 µmol/L on diet alone) but is not yet available in Saudi Arabia. Its immunogenic profile [56]—which requires immunomodulation protocols and careful dose titration—and its restriction to adults limit its current utility.

### 7.3. Emerging and Investigational Therapies

Gene therapy is the most transformative potential advancement in PKU management. Preclinical studies using adeno-associated virus (AAV) vectors to deliver a functional PAH gene to hepatocytes have evidenced normalization of phenylalanine in murine PKU models [57]. Phase I/II human trials are ongoing, and preliminary data suggest durable phenylalanine reductions after a single administration [58]. Key challenges include immune responses to the AAV vector, uncertainty about long-term transgene expression, potential genotoxicity [59], and suitability for pediatric populations, in whom liver growth and vector dilution may limit durability. mRNA-based therapy, which delivers PAH mRNA encapsulated in lipid nanoparticles to the liver, is an alternative gene-directed strategy that avoids the genomic integration concerns associated with AAV. Because mRNA is transient, repeat dosing would be required; however, lipid nanoparticle technology has advanced substantially since the COVID-19 vaccine program. Preclinical studies in Pahenu2 PKU mice have shown significant phenylalanine reductions with repeated mRNA-LNP dosing, while human trials are still at an early stage.

CRISPR-based and base-editing approaches to correct pathogenic PAH variants at the genomic level are in preclinical development. These technologies offer the possibility of a single-administration curative therapy but face major barriers, including off-target editing risk, delivery to enough hepatic tissue, and the high allelic heterogeneity of the PAH gene, which makes a universal editing strategy technically difficult.

### 7.4. Saudi Case-Level Evidence for Pegvaliase

Although pegvaliase remains formally unavailable in Saudi Arabia and is approved elsewhere only for adults, Alfadhel and Albarakati reported its off-label use in a 12-year-old Saudi girl with uncontrolled PKU who was monitored for one year [60]. A therapeutic response became evident at six months, with phenylalanine stabilizing in the <5–14 µmol/L range on an unrestricted diet, and no major safety concerns were reported during the observation period. As a single pediatric case rather than a clinical trial, this report cannot prove pediatric safety or efficacy, but it is, to date, the only published Saudi experience with pegvaliase in a child and offers an early real-world signal that may inform future discussions about expanded pediatric access.

## 8. Emerging Research and Precision Medicine

The concept of precision medicine—delivering the right therapy to the right patient based on individual biological characteristics—is especially well suited to PKU, where genotype reliably predicts BH4 responsiveness, phenotype severity correlates with residual enzyme activity, and emerging therapies have mutation-specific mechanisms of action. The clinical translation of precision medicine in PKU requires three foundational elements: comprehensive genotyping of all patients, functional characterization of variant-specific PAH enzyme activity (in vitro or through bioinformatic prediction), and systematic BH4 loading test data linked to genotype.

In the Saudi context, the path to precision PKU medicine begins with the characterization of the national PAH mutation landscape. A national registry linking genotype, phenotype, BH4 responsiveness, and long-term outcome data would enable genotype-driven treatment algorithms similar to those increasingly used in European PKU centers. Bioinformatic tools, including PolyPhen-2, SIFT, and PAH-specific variant effect predictors, can provide functional predictions for novel variants, but experimental validation in cell models or patient-derived hepatic organoids is ultimately required for clinical-grade classification. Beyond single-variant effect prediction, emerging computational frameworks that integrate multiplex biological network dynamics offer a complementary approach to disease–gene association, modeling genes and their regulatory and protein-interaction relationships as interconnected network layers to prioritize candidate genes and disease modifiers that single-variant tools cannot capture [61]. Related advances in self-supervised and denoising machine learning for disease–gene association prediction, which learn robust gene and disease representations from large-scale biological data while filtering noisy auxiliary signals, are directly relevant to the genotype–phenotype correlation challenges described for PAH variants, where phenotypic labels are often incomplete or inconsistently reported across cohorts [62]. Applying these network- and learning-based approaches to the PAH variant landscape could help prioritize candidate disease-modifier genes underlying the phenotypic variability observed even among patients sharing the same PAH genotype, an open question in Saudi and other consanguineous populations where genetic background may differ systematically from outbred cohorts. Complementary analytical advances are also emerging in the broader diagnostic landscape: spectroscopic platforms combined with machine learning classifiers, for example vibrational spectroscopy paired with deep-learning models, have shown high sensitivity for detecting biochemical signatures of metabolic dysfunction and may hold future translational relevance for rapid confirmatory testing or longitudinal monitoring in rare inborn errors of metabolism such as PKU, particularly where mass spectrometry infrastructure is limited [63].

Multi-omic approaches—integrating metabolomics, proteomics, and transcriptomics with genomic data—offer opportunities to identify biomarkers of neurological risk and treatment response beyond plasma phenylalanine. Neurocognitive biomarkers—including brain Phe quantification by 19F-MRS, CSF neurotransmitter metabolites, and functional neuroimaging—are being explored in research settings as more sensitive indicators of CNS phenylalanine burden than blood levels alone. These approaches are not yet clinically available in Saudi Arabia, but they represent an important direction for translational research.

Telemedicine and digital health platforms are increasingly relevant to PKU care, particularly in Saudi Arabia, where geographic distance and regional healthcare disparities create barriers to specialist access. Remote dietary counseling, digital Phe monitoring through dried blood spot home testing, and smartphone-based food tracking applications can support adherence, especially in adolescents and young adults, the age group at highest risk of dietary dropout. Integrating digital tools with national PKU registry platforms could improve surveillance, real-world data collection, and individualized clinical support.

Several concrete policy measures deserve priority in Saudi Arabia. First, pre-marital carrier screening for PAH variants should be formally integrated into existing premarital testing programs, which already include screening for hemoglobinopathies and glucose-6-phosphate dehydrogenase deficiency, to identify at-risk couples before conception and support informed reproductive decision-making. Second, a national PKU patient registry linking newborn screening data with longitudinal genotype, dietary management, metabolic control, and neurodevelopmental outcome records should be established as an institutional priority; such a registry would provide the evidence base for Saudi-specific clinical guidelines and allow for meaningful comparisons with international cohort data. Third, all neonates should receive timely screening within 48–72 h of birth, and healthcare providers, including metabolic dietitians and primary care physicians, should receive updated training on low-phenylalanine dietary management, amino acid formula access, and current pharmacological options. Together, these measures would substantially reduce preventable morbidity from PKU across the Kingdom.

## 9. Saudi Arabia: Challenges and Future Directions

Despite a functioning newborn screening program, Saudi Arabia faces a complex set of challenges in delivering equitable, high-quality PKU care to all affected individuals across the Kingdom [Table 5]. These challenges span epidemiological, genetic, clinical, logistical, and health-policy domains, and addressing them will require coordinated action from clinicians, researchers, health authorities, and patient advocacy organizations.

The absence of a national PKU registry is arguably the most significant structural gap. Without systematic data on incidence, genotype distribution, treatment adherence, metabolic control, and neurodevelopmental outcomes, evidence-based policy cannot be formulated and quality improvements cannot be measured. Establishing a national registry linked to the existing newborn screening program and modeled on successful registries in Germany, the United Kingdom, and Turkey should therefore be a short-term priority. Such a registry would also facilitate international collaborative research and allow Saudi Arabia to contribute to global datasets of PKU natural history.

Medical formula access is a persistent practical challenge. The specialized low-phenylalanine amino acid formulas and medical foods required for PKU dietary management are mainly manufactured abroad and are subject to import costs, supply-chain disruptions, and inconsistent reimbursement. Developing locally manufactured PKU formula, potentially in partnership with Saudi Arabia’s expanding pharmaceutical and food technology sectors, would improve availability, reduce costs, and enhance cultural acceptability, including through the availability of Halal-certified products.

Transition of care from pediatric to adult metabolic services is a recognized challenge in PKU management globally, and Saudi Arabia is no exception [Table 5]. Many patients lose specialist contact after childhood, dietary compliance declines, and metabolic control worsens. Structured transition programs, adult metabolic clinics with multidisciplinary expertise, and community-based dietitian services are needed to sustain care quality across the lifespan.

Maternal PKU—the teratogenic consequence of elevated phenylalanine during pregnancy—is particularly significant in the Saudi context, where PKU prevalence is high and unplanned pregnancies may occur in women who have disengaged from follow-up. Pre-conception counseling, early identification of reproductive-age women with PKU in primary care, and access to intensified metabolic management during pregnancy are essential components of a comprehensive PKU care system.

## 10. Conclusions

This review has synthesized current evidence on PKU epidemiology, pathophysiology, genetics, diagnosis, and therapeutics with particular emphasis on the Saudi Arabian context. The evidence base reveals a condition of high clinical and public health relevance in Saudi Arabia, shaped by founder-effect mutation enrichment, high consanguinity, and significant structural gaps in registry infrastructure, BH4 responsiveness data, and specialist access. Addressing these gaps through the priorities outlined in Section 8 and Section 9 of this review represents the most direct path to improving outcomes for patients and families living with PKU in the Kingdom.

As precision medicine transforms the management of rare metabolic diseases globally, Saudi Arabia is well positioned to contribute to and benefit from these advances, provided that the foundational infrastructure of data, access, and multidisciplinary care is put in place. This review is intended to support this effort and to serve as a starting point for clinicians, researchers, and policymakers committed to improving outcomes for children and families living with PKU in Saudi Arabia.

## Figures and Tables

**Table 1:** PKU Epidemiology and Saudi Context. Data compiled from van Spronsen et al. [2], Shoraka et al. [10], and published Saudi cohort studies cited in the text; specific per-cell citations are provided where a single source directly supports that figure.

Clinical Relevance	Saudi Arabia	Global Estimate	Parameter
Among the highest worldwide; consanguinity-driven	~18–24 (estimate)	~6.0 (pooled meta-analysis estimate) [10]	Incidence (per 100,000 live births)
High allele frequency in population	Not precisely established; presumed higher than global average given consanguinity rate	1 in 50 (global)	Carrier frequency
Genotype–phenotype correlation relevant	R252W (most prevalent, 26.4% in largest cohort), R261Q, IVS10-11G>A, novel variants	Varied by ethnicity	Most common mutation type
Increases homozygosity; recessive risk elevated	~50–60% (Saudi)	<1% (Western)	Consanguinity rate
Early detection; outcome improvement possible	National NBS since 1980s (expanded)	Routine in >50 countries	Newborn screening status
Higher dietary and medication burden	Classic PKU predominant in cohorts	Mild/moderate/classic	PAH deficiency severity
Major gap in Saudi PKU outcome research	Variable; limited local data	~30–60% (Europe)	Dietary compliance rate (adult)
Precision therapy opportunity	Understudied in Saudi population	~20–50% (mild/moderate)	BH4 responsiveness

**Table 2:** Major PAH variants and genotype–phenotype associations. Variant data compiled from Hillert et al. [20], Pey et al. [22], Al-Sayed et al. [23], and Balobaid et al. [24]; Saudi-specific frequency data compiled from Balobaid et al. [24] and Al-Sayed et al. [23].

Notes/Saudi Relevance	BH4 Response	PKU Class	Type	PAH Variant
Most common globally; severe phenotype; reported in Saudi cohorts	None	Classic PKU	Missense	p.R408W (c.1222C>T)
Reported in Saudi and Arab populations; BH4-responsive	Positive (~60%)	Mild/moderate PKU	Missense	p.R261Q (c.782G>A)
Common in Middle East; leads to exon skipping	None/poor	Classic PKU	Splicing	IVS10-11G>A (c.1066-11G>A)
Found in Arab populations; intermediate severity	Variable	Moderate PKU	Missense	p.Y414C (c.1241A>G)
Associated with mild hyperphenylalaninemia; BH4-responsive	Positive	Mild PKU/MHP	Missense	p.I65T (c.194T>C)
Rare; loss of function; no sapropterin benefit	None	Classic PKU	Missense	p.L48S (c.143T>C)
Most prevalent variant in largest published Saudi cohort to date (26.4% of alleles)	Variable	Classic/moderate PKU	Missense	p.R252W (c.754C>T)
Saudi Arabia has a high rate of novel variants due to consanguinity; WES recommended	Unknown	Variable	Various	Novel/private variants
Less common in consanguineous population; phenotype predicts from null allele	Allele-dependent	Variable	Mixed alleles	Compound heterozygotes

**Table 4:** Therapeutic options and indications. Compiled from published guidelines and the primary literature [43,44,45].

Saudi Availability	Limitations	Indication	Mechanism	Therapy
Partially available; import-dependent	Palatability; compliance; cost; formula access	All PKU; lifelong in classic; relaxed in mild	Reduce Phe intake; supplement essential AA	Phe-restricted diet + formula
Approved; limited coverage	Non-responders (~50–80% of classic); expensive	BH4-responsive PKU (mild–moderate, select mutations)	Pharmacological BH4—stabilizes PAH enzyme	Sapropterin (Kuvan^®^)
Not yet available in KSA	Immunogenic; injection burden; not pediatric	Adults with uncontrolled classic PKU	Subcutaneous PAL enzyme substitution	Pegvaliase (Palynziq^®^)
Limited; specialist-prescribed	No Phe lowering; brain-level effect only	Adjunct; adult PKU with poor diet adherence	Compete with Phe at BBB transporters	Large neutral amino acids (LNAA)
Not available	Experimental; durability unknown; immune response	Classic PKU; Phase I/II trials	AAV-mediated PAH gene restoration	Gene therapy (investigational)
Not available	Very early stage; repeat dosing needed	Preclinical/early Phase I	Hepatic delivery of PAH mRNA	mRNA therapy (investigational)
Very limited locally	Limited formula options; not universal	Children and adults; diet palatability	Low-Phe protein source from whey	Glycomacropeptide (GMP) diet

**Table 5:** PKU management in Saudi Arabia challenges and future directions.

Proposed Future Direction	Current Challenge	Domain
Establish Saudi PKU registry; publish national cohort data	Limited published incidence data; no national PKU registry	Epidemiology
National PAH mutation database; WES for novel variant discovery	Spectrum of PAH variants not fully characterized in Saudi population	Genetic landscape
Standardize NBS-to-clinic pathway; improve rural access	NBS exists but follow-up infrastructure varies by region	Newborn screening
Support local low-Phe food production; subsidy programs	Medical formula largely imported; palatability barriers; no local GMP formula	Dietary management
National reimbursement policy; regulatory pathway for new therapies	Sapropterin approved but reimbursement limited; pegvaliase unavailable	Treatment access
National PKU clinic protocol; longitudinal neurodevelopmental data	No standardized follow-up protocol; cognitive outcomes under published	Neurodevelopmental monitoring
PKU-specific dietitian training; patient support groups; telehealth	Diet adherence poor in adolescents/adults; limited psychosocial support	Patient adherence
Mandatory pre-conception PKU counseling; maternal PKU clinics	Underrecognized; high-risk pregnancies with poor metabolic control	Maternal PKU
Collaborative Gulf/Arab PKU research network; grant funding for precision medicine	Few Saudi PKU-specific publications; limited translational research	Research capacity
Systematic BH4 loading test protocols; functional variant classification	BH4 responsiveness data limited; no genotype–phenotype database	Precision medicine

## Data Availability

No new data were created or analyzed in this study. Data sharing is not applicable to this article.
